# Supplementary material for: From sequence to enzyme mechanism using multi-label machine learning
Source: BMC Bioinformatics. 2014 May 19;15:150. doi: 10.1186/1471-2105-15-150 (PMC4229970; doi:10.1186/1471-2105-15-150)
Supplement: Additional file 2 — Java code of ml2db. Additional file ml2db_code.tar.gz contains the Java source code to run the multi-label machine learning experiments and save the results to database. The code’s Javadoc is included. [file 1471-2105-15-150-S2.zip › additional file 2/ml2db/ml2db/doc/mulan/transformations/multiclass/package-use.html]

Uses of Package mulan.transformations.multiclass


JavaScript is disabled on your browser.


- Overview
- Package
- Class
- Use
- Tree
- Deprecated
- Index
- Help

- Prev
- Next

- Frames
- No Frames

- All Classes

# Uses of Package mulan.transformations.multiclass

- Packages that use mulan.transformations.multiclass

  | Package | Description |
  | --- | --- |
  |  |  |
  | --- | --- |
  | mulan.classifier.transformation |  |
  | mulan.dimensionalityReduction |  |
  | mulan.transformations.multiclass |  |
- Classes in mulan.transformations.multiclass used by mulan.classifier.transformation

  Class and Description

  MultiClassTransformation

  The interface for single-label multi-class transformations.
- Classes in mulan.transformations.multiclass used by mulan.dimensionalityReduction

  Class and Description

  MultiClassTransformation

  The interface for single-label multi-class transformations.
- Classes in mulan.transformations.multiclass used by mulan.transformations.multiclass

  Class and Description

  Copy

  Class that implement the Copy transformation method

  MultiClassTransformation

  The interface for single-label multi-class transformations.

  MultiClassTransformationBase

  The base class for multi-class transformation methods.

  SelectionType

- Overview
- Package
- Class
- Use
- Tree
- Deprecated
- Index
- Help

- Prev
- Next

- Frames
- No Frames

- All Classes
